# The whole-genome sequence of *Bacillus* sp. KICET-3, isolated from doenjang

**DOI:** 10.1128/mra.01190-23

**Published:** 2024-04-22

**Authors:** Hyojung Park, Hye Sun Lee, Jin Hyung Lee, Byoung Seung Jeon

**Affiliations:** 1 Biomaterials and Processing Center, Korea Institute of Ceramic Engineering and Technology, Cheongju-si, Chungcheongbuk-do, South Korea; Wellesley College, Wellesley, Massachusetts, USA

**Keywords:** *Bacillus*, Doengjang, Antioxidant effect

## Abstract

*Bacillus* sp. KICET-3, isolated from doenjang, a traditional Korean fermented food, has a single chromosomal DNA fragment of 4,616,861 bp, and the G+C content is 45.52%. It is estimated to have 4,450 predicted coding DNA sequences, 84 tRNAs, and 24 rRNAs.

## ANNOUNCEMENT

Among the many benefits of doenjang, its antioxidant effect is a well-established function (1). Among the various isolates from doenjang (Cheongju-si), KICET-3 is an isolate whose antioxidant effect was confirmed through a 2,2-diphenyl-1-picrylhydrazyl assay (2). KICET-3 was isolated by spreading doenjang diluted with water on tryptic soy agar containing skim milk and culturing at 30°C for 48 hours. This strain formed yellow colonies and exhibited the ability to decompose skim milk. Based on 16S rRNA sequencing of KICET-3 using the universal primers 27F (5′-AGAGTTTGATCMTGGCTCAG-3′) and 1492R (5′-TACGGYTACCTTGTTACGACTT-3′) (3), it was 99% identical to *Bacillus sonorensis* NBRC 101234^T^ (AYTN01000016).

A single colony of KICET-3 was inoculated into tryptic soy broth and cultured at 30°C and 150 rpm for 48 hours. The cells were harvested from the culture broth, after which genomic DNA (gDNA) was extracted with the Maxwell 16 Cell DNA Purification Kit (Promega, USA). The gDNA was sequenced on both the Illumina HiSeq X Ten platform (151-bp paired-end reads) and the PacBio Sequel system by Macrogen Inc. (Seoul, Republic of Korea). For Illumina sequencing, libraries were prepared using TruSeq Nano DNA high-throughput (Illumina) library preparation kits. From the total of 19,176,566 reads, 9,178,508 reads were obtained after filtering, and these were used for analysis. For quality control of the Illumina data, Q30 reads were filtered by ≤90%, after which adapter trimming and read filtering were performed by Trimmomatic v.0.38 (options: ILLUMINACLIP:Adapter:fasta:2:30:10:8:true LEADING:15 TRAILING:15 SLIDINGWINDOW:4.15 MINLEN:36) (4). Using high-quality adapter-trimmed Illumina readouts, the assembly was polished in triplicate with Pilon v.1.21 (5). The reads generated from the Illumina system were used to correct errors in the data where assembly was performed using subreads generated from the PacBio system. The HiFi libraries were prepared with the SMRTbell Express Template Prep Kit 2.0 (PacBio) for PacBio sequencing. The gDNA was sheared by a Megaruptor 3 (Diagenode, USA), and size selection was performed using the BluePippin size selection system (cutoff value: <8 kb) after library production. The PacBio Sequel data comprised 109,723 reads and 921,526,515 bp (*N*_50_: 9,073 bp). Subreads generated from the PacBio Sequel system were assembled using Flye v.2.4.2, and the assembly was polished using Racon v1.4.21 (6). Assessment of the quality of preassembled reads was performed using BLAST+ v.2.7.1+ with the NCBI database. After the genome was assembled, NCBI Prokaryotic Genome Annotation Pipeline v.6.6 was used to perform gene prediction and functional annotation (7). Default parameters were used for all software unless otherwise specified.

The *de novo* assembly generated a single chromosome with a G+C content of 45.5%, an estimated genome size of 4,616,861 bp, and an average reference coverage of 199.4×. When the two ends of a contig overlapped, the contig was regarded as circular; if there was no overlap, the contig might have been linear originally or there might have been gaps at the end of the contig. Through Flye analysis, the genome was defined as one circular chromosome. The whole genome contained 4,695 identified genes, with 4,450 predicted coding DNA sequences, 83 tRNAs, and 24 rRNAs.

## Data Availability

The whole-genome sequencing data for *Bacillus* sp. KICET-3 were assigned the GenBank accession number CP137345, 16S rRNA accession number OR807426, BioProject accession number PRJNA1031784, and BioSample accession number SAMN37973075, and the SRA numbers are SRR2664315 and SRR2664316. This strain was deposited in the Korean Culture Center of Microorganisms (KCCM) under accession number KCCM 13299P.

## References

[1] Lee S, Lee Y-B, Lee C-H, Park I. 2021. Effects of the addition of herbs on the properties of doenjang. Foods 10:1307. doi:10.3390/foods10061307 34200252 PMC8227189

[2] Kedare SB, Singh RP. 2011. Genesis and development of DPPH method of antioxidant assay. J Food Sci Technol 48:412–422. doi:10.1007/s13197-011-0251-1 23572765 PMC3551182

[3] Frank JA, Reich CI, Sharma S, Weisbaum JS, Wilson BA, Olsen GJ. 2008. Critical evaluation of two primers commonly used for amplification of bacterial 16S rRNA genes. Appl Environ Microbiol 74:2461–2470. doi:10.1128/AEM.02272-07 18296538 PMC2293150

[4] Bolger AM, Lohse M, Usadel B. 2014. Trimmomatic: a flexible trimmer for Illumina sequence data. Bioinformatics 30:2114–2120. doi:10.1093/bioinformatics/btu170 24695404 PMC4103590

[5] Walker BJ, Abeel T, Shea T, Priest M, Abouelliel A, Sakthikumar S, Cuomo CA, Zeng Q, Wortman J, Young SK, Earl AM. 2014. Pilon: an integrated tool for comprehensive microbial variant detection and genome assembly improvement. PLoS One 9:e112963. doi:10.1371/journal.pone.0112963 25409509 PMC4237348

[6] Kolmogorov M, Yuan J, Lin Y, Pevzner PA. 2019. Assembly of long, error-prone reads using repeat graphs. Nat Biotechnol 37:540–546. doi:10.1038/s41587-019-0072-8 30936562

[7] Li W, O’Neill KR, Haft DH, DiCuccio M, Chetvernin V, Badretdin A, Coulouris G, Chitsaz F, Derbyshire MK, Durkin AS, Gonzales NR, Gwadz M, Lanczycki CJ, Song JS, Thanki N, Wang J, Yamashita RA, Yang M, Zheng C, Marchler-Bauer A, Thibaud-Nissen F. 2021. RefSeq: expanding the prokaryotic genome annotation pipeline reach with protein family model curation. Nucleic Acids Res 49:D1020–D1028. doi:10.1093/nar/gkaa1105 33270901 PMC7779008

