# Erratum for Park et al., “The whole-genome sequence of *Bacillus* sp. KICET-3, isolated from doenjang”

**DOI:** 10.1128/mra.00309-25

**Published:** 2025-06-18

**Authors:** Hyojung Park, Hye Sun Lee, Jin Hyung Lee, Byoung Seung Jeon

## ERRATUM

Volume 13, no. 6, e01190-23, 2024, https://doi.org/10.1128/mra.01190-23. Page 2, Acknowledgments: “...(1415185878, Development of biomass-derived solvent grade methyltetrahydrofuran production and separation and purification process) funded By the Ministry of Trade, Industry & Energy(MOTIE, Korea)” should read “…(grant no. 1415185928, Development of MTHF production technology from intermediates [levulinic acid, furfural] and evaluation of MTHF solvent applicability) funded by the Ministry of Trade, Industry & Energy (MOTIE, Korea).”

